# Lung Function Impairment Among Paint Factory Workers: A Cross-Sectional Analytical Study

**DOI:** 10.7759/cureus.68499

**Published:** 2024-09-03

**Authors:** Stella I Omogbeme, Uchechukwu O Agboje, Emmanuel Obazee

**Affiliations:** 1 Internal Medicine, Delta State University Teaching Hospital, Oghara, NGA; 2 Respiratory Medicine, New Cross Hospital, Wolverhampton, GBR; 3 Internal Medicine, University of Port Harcourt Teaching Hospital, Port Harcourt, NGA

**Keywords:** cross-sectional study, occupational hazards, volatile organic compounds, paint, lung impairment, lung function test

## Abstract

Background: A paint factory or manufacturing is a vocation characterized by exposure to chemical hazards during production. Paint exposure plays a great role in the incidence of several health problems, particularly respiratory diseases. The study aims to assess the pattern of spirometric indices among the study population.

Methods: This cross-sectional study of paint factory workers (PFWs) assesses their spirometric findings in Delta State, Nigeria. The participants for this study were divided into two groups; the PFWs and the non-PFWs (NPFW) which serve as the control group. Hundred and 200 participants were recruited for the study group and control, respectively.

Results: Among the participants in the paint worker cohort, 45 individuals (45.0%) had been employed for less than five years while 14 (14.0%) had worked over 10 years. Eighty-eight (88%) are aware of PPE; however, only 12 (12.0%) use them always. Findings show that 178 (89.0%) vs. seven (7.0%) of NPFW and PFW had normal pulmonary function tests. The spirometric abnormalities within the PFW group were obstructive lung disease affecting 59 (59.0%) of the cohort while 34 (34.0%) had restrictive lung patterns.

Conclusion: Exposure to volatile organic compounds (VOCs) emitted from paint fumes is associated with spirometric abnormalities with obstructive patterns more predominant than restrictive patterns.

## Introduction

Paint is any pigmented liquid, liquefiable, or solid resin composition that, after application to a surface in a thin stratum, alters to a solid film [1]. Benzene, xylene, and toluene are the most common volatile organic compounds (VOCs) used in the formulation of paints. Exposure to paint is found to play a great role in the incidence of several health problems, particularly respiratory diseases [2]. Considerable inhalation and percutaneous absorption of these VOC can occur within minutes of the onset of exposure [3].

Paint factory workers (PFWs) work mainly in the small and medium-scale sectors of the economy, and this is a vocation characterized by exposure to chemical hazards during the paint manufacturing system. Studies assessing the health effects of exposure to VOCs are mainly from developed countries [4]. The prevalence of adverse health effects as a result of organic solvents used among PFWs in Nigeria is not well documented. In Nigeria, particularly in Delta State, PFWs are exposed to these VOCs in the paint during crushing, grinding, and mixing [5]. During mixing, the various paint components such as paint pigments, binders, and solvents are mixed in their right proportion. This mixing process is done with the use of personal protective equipment (PPE) such as gloves, aprons, nose masks, etc., by the PFWs [6].

Clean air is a fundamental necessity for life. In recent years, advancements in occupational health, safety, and hygiene knowledge have significantly enhanced workplace conditions, thereby reducing workers' exposure to multiple toxic substances in various factories [7]. WHO defines air pollution as material presence in the air of the working environment causing harmful effects to workers at any given time, they come into contact beyond the threshold concentration levels of the substance [5]. The degree of chemicals released into the air correlates with the different units in the paint factories and should not exceed the WHO permissible standard units [8]. The exposure limit can be of three forms: time-weighted average (TWA), permissible exposure limits (PEL), or short-term exposure limit (STEL). TWA is the average airborne concentration of a particular substance when calculated over a normal eight-hour working day, for a five-day working week [9]. The Nigerian national ambient air quality standards stipulate 6,000µg/m^3^ (1.9 ppm) per day as a permissible limit for total VOCs in a workplace environment [10].

Health hazards among PFWs are among the challenges faced by both developed and developing nations. The actual burden arises from difficulty in determining the precise particles of the industrial products that cause these diseases in man. The impact of these industrial products on the factory workers is multi-systemic, with the respiratory system being one of the major target organs as direct inhalation of these products from prolonged exposure is capable of causing harm. The major challenge in detecting the effect of paint on an individual is the respiratory changes that occur over a long period and from repeated exposure. Therefore, measuring the amount that is likely to cause pathological changes poses a challenge in developing countries.

There is a scarcity of research in Nigeria concerning exposure to VOCs in paint and its impact on the respiratory system. In Delta State, insufficient attention has been given to this occupational and environmental health issue. Therefore, this study aims to assess the pattern of spirometric indices among PFWs and identify the associated factors.

## Materials and methods

Study area

This study was carried out among PFWs in Delta State, Nigeria. Delta State is located in the South-South geopolitical zone of Nigeria with the capital city at Asaba. It is one of the most populous and well-known cities in Delta State. It has 25 local government areas with rapid urbanization taking place in these major areas. The paint factories are located in all three senatorial districts in Delta State: Delta Central (Sapele and Ughelli), Delta South (Warri), and Delta North (Obiaruku, Agbor, and Asaba).

Study design

This study was a cross-sectional study of PFWs to assess their spirometric findings. The participants for this study were divided into two groups; the PFWs and the non-PFWs (NPFW) which was the control group.

Study population/sample size estimation

The study population comprised all PFWs across different parts of Delta State where a total of 100 persons were recruited for the study. Two hundred (200) control participants were also recruited. The ratio of the participants to control was 1:2. The selected factory workers (100) were from the three Senatorial districts of Delta State; Delta Central (44), Delta North (41), and Delta South (35).

Subject selection/sampling

Following randomization, two factories in Sapele (with 34 workers), one factory in Ughelli (with 10 workers), three factories in Warri (with 35 workers), one factory in Obiaruku (with six workers), one factory in Agbor (with nine workers), and three factories in Asaba (with 26 workers). Selections of these factories were done using the simple random sampling technique. The locations of the factories were identified by reference to the list of paint factories prepared by the Association of Paint Manufacturers in Delta State. A simple random sampling method was used in the selection of subjects to be recruited in each factory. This technique takes a small random portion of the entire population to represent the entire data set, with each member having an equal probability of being selected. Only consenting subjects who satisfy the conditions under the inclusion criteria were recruited. The process involved cutting up small pieces of paper which corresponded to the number of staff in each paint factory. A unique identifier was then made on each paper up to the total number of papers which equaled the required number of participants in each factory - the rest were left blank. The pieces of paper were subsequently folded to conceal the unique identifiers. Participants were then asked to pick a single paper - those who picked the papers containing the unique identifiers were recruited into the study while those who picked the blank papers were not recruited. Any participant who picked a paper with the identifier but did not meet the inclusion criteria was replaced with another staff using simple balloting from the pool of others.

Inclusion criteria

Inclusion criteria include the following, PFWs between the ages of 18 and 60 years, those who can do spirometry maneuvers, and those who gave informed consent.

Exclusion criteria

Participants meeting the following criteria were excluded from the study: PFWs unable to perform spirometry maneuvers; those presenting with chest deformities; individuals with co-morbid conditions such as cardiac, renal, or hepatic diseases, as determined by their medical history; subjects taking medications, including codeine or psychoactive drugs; those with known respiratory diseases, such as asthma, chronic obstructive pulmonary disease (COPD), or other respiratory disorders; and current or former smokers.

Selection of controls

Controls for the study were selected from workers who had never worked in a paint factory and those who lived about 2 km or more away from the factory. They were matched for age and sex. Informed consent was obtained from the control subjects.

Study instruments

A questionnaire was developed to obtain relevant sociodemographic and work-related information, as well as for documentation of spirometric findings of study participants. Relevant sociodemographic and work-related information includes the factory site (if applicable), age, sex, tribe, marital status, occupation, religion, highest level of education, Duration of employment, nature of work, awareness of PPEs, and use of PPEs. Spirometric finding indices include a recorded value of FEV1 (L), forced vital capacity (FVC) (L), FEV1/FVC (%), PEFR (L/min), and FEF 25%-75%. The MIR Spirolab Spirometer (Medical International Research, Italy) is given in Appendices.

Spirometry test

Using the open circuit method, participants were asked to sit upright in a chair with legs uncrossed and feet flat on the ground. He or she was asked to breathe in completely and rapidly, after which a pause for about one second was observed. The subject then placed his/her mouth on the mouthpiece of the spirometer with the lips closed around it to form a good seal. The test was carried out per ATS/ERS acceptability and repeatability criteria. Predicted values were determined for each subject and abnormal results for FEV1, FVC, and FEV1/FVC were determined by comparison to their lower limits of normal (LLN).

A forced expiratory volume in one second > or < 80%, FVC > 80%and FEV1/FVC < 70% were used to define obstructive lung disease, restrictive lung disease was defined as forced expiratory volume in one second < 80%, FVC < 80% and FEV1/FVC ratio of > 70% was used. While mixed pattern of disease was defined as FVC < 80%, FEV1 < 80%, and (FEV1/FVC) < 70%.

Ethical approval

Ethical approval was sought and obtained from the Health Ethics and Research Committee of the Delta State University Teaching Hospital, Oghara, with approval number HREC/PAN/2020/029/0372 Participants were assured of confidentiality and they participated at no cost as all the financial obligations were borne by the researcher.

Data analysis

The data were analyzed using the IBM Statistical Package for Social Sciences (SPSS) program Version 26.0 (IBM Corp., Armonk, NY). Descriptive statistics for categorical variables including age, sex, marital status, educational status, religion, and ethnic group were summarized as frequencies and percentages Inferential statistics were done using a Chi-squared test and student t-test. A p-value of <0.05 was considered to be statistically significant.

## Results

Table 1 demonstrates the socio-demographic characteristics of the study participants. Thirty (30.0%) study participants in the PFW group and 60 study participants in the NPWF group were from Warri, while 27 (27.0%) were from the PFW group and 54 (27.0%) NPFW group were from Sapele town. This difference was not statistically significant (p = 1.000).

**Table 1:** Socio-demographic characteristics of the study participants PFW - paint factory workers, NPFW - Non-paint factory workers, ꭓ2 - Chi-square test, F - Fishers Exact Test, P-values <0.05 are significant

Variable	PFW group	NPFW group	Total Freq. (%)	Test statistics	P-value
	Freq. (%) (n=100)	Freq. (%) (n=200)			
Location					
Warri	30 (30.0)	60 (30.0)	90 (30.0)	ꭓ2=0.0001	1.000
Sapele	27 (27.0)	54 (27.0)	81 (27.0)		
Asaba	22 (22.0)	44 (22.0)	66 (22.0)		
Ughelli	8 (8.0)	16 (8.0)	24 (8.0)		
Agbor	8 (8.0)	16 (8.0)	24 (8.0)		
Obiaruku	5 (5.0)	10 (5.0)	15 (5.0)		
Age group (Years)					
< 20	1 (1.0)	10 (5.0)	11 (3.7)	ꭓ2= 4.038	0.405
20-29	30 (30.0)	54 (27.0)	84 (28.0)		
30-39	23 (23.0)	49 (24.5)	72 (24.0)		
40-49	31 (31.0)	52 (26.0)	83 (27.7)		
>50	15 (15.0)	35 (17.5)	50 (16.6)		
Mean Age (Years)	37.3 ± 10.9	36.8 ± 11.9	37.0. ± 11.3	t =-0.346	0.730
Sex					
Male	57 (57.0)	108 (54.0)	165 (55.0)	ꭓ2= 0.242	0.712
Female	43 (43.0)	92 (46.0)	135 (45.0)		
Marital status					
Single	47 (47.0)	104 (52.0)	151 (50.3)	ꭓ2= 0.885	0.643
Married	48 (48.0)	89 (44.5)	137 (45.7)		
Widowed	5 (5.0)	7 (3.5)	12 (4.0)		
Educational level					
No formal	1 (1.0)	11 (5.5)	12 (4.0)	ꭓ2= 3.568	0.322
Primary	18 (18.0)	35 (17.5)	53 (17.7)		
Secondary	48 (48.0)	89 (44.5)	137 (45.7)		
Tertiary	33 (33.0)	65 (32.5)	98 (32.6)		
Religion					
Christian	97 (97.0)	192 (96.0)	289 (96.3)	F = 0.382	0.876
Muslim	2 (2.0)	6 (3.0)	8 (2.7)		
Atheist	1 (1.0)	2 (1.0)	3 (1.0)		
Ethnic group					
Igbo	25 (25.0)	51 (25.5)	76 (25.3)	ꭓ^2^= 1.733	0.887
Benin	19 (19.0)	30 (15.0)	49 (16.3)		
Urhobo	18 (18.0)	38 (19.0)	56 (18.7)		
Ika	17 (17.0)	44 (22.0)	61 (20.3)		
Isoko	16 (16.0)	28 (14.0)	44 (14.7)		
Yoruba	5 (5.0)	9 (4.5)	14 (4.7)		

A higher proportion, 31.0% (n=31) of study participants in the paint worker group and 26.0% (n=52) in the non-paint worker group belong to the 40 to 49 years age group. This difference was not statistically significant (p = 0.405)

Fifty-seven (57.0%) and 108 (54.0%) study participants in the paint worker and non-paint worker groups respectively were male. This difference was not statistically significant (p = 0.712). Forty-eight (48.0%) and 89 (44.5%) study participants in the paint worker group and non-paint worker group respectively were married. This difference was not statistically significant (p = 0.643).

Forty-eight (48.0%) and 89 (44.5%) study participants in the paint worker group and non-paint worker group respectively had attained the secondary level of education. This difference was not statistically significant (p = 0.322).

Ninety-seven (97.0%) and 192 (96.0%) study participants in both the paint worker group and non-paint worker group respectively were Christians. This difference was not statistically significant (p = 0.876). A higher proportion of study participants in both the paint worker group, 25.0% (n=25), and the non-paint worker group, 25.5% (n=51) were from the Igbo ethnic group (p = 0.887).

Table 2 shows the duration of occupation of PFWs. Most of the study participants in the paint worker group, 45 (45.0%) worked for less than five years; while the least proportion, 14 (14.0%), had worked for more than 10 years. The mean (SD) duration of work in the paint worker group is 6.0 ± 3.4 years.

**Table 2:** Duration of occupation of paint factory workers study participants PFW - paint factory workers

Duration	PFW group (n=100)	
	Frequency	Percent
≤5 years	45 (45.0)	45.0
6-10 years	41 (41.0)	41.0
>10 years	14 (14.0)	14.0
Mean ± SD (years)	6.0 ± 3.4	

In Table 3, the awareness of, usage, and frequency of use of personal protective devices during work by paint factory study participants are represented. Eighty-eight (88.0%) PFWs were aware of personal protective devices; 81 (81.0%) had previously used any form of PPEs while 12 (14.9%) used it always, 21 (25.9%) used it sometimes and 18 (22.2%) rarely used them. Twenty-one (25.9%) of PFWs reported using the PPEs only when a supervisor was present.

**Table 3:** Awareness of usage and frequency of use of personal protective devices during work by paint factory study participants PPE - Personal protective equipment

Variable	Frequency (n=100)	Percent
Awareness of personal protective devices		
Yes	88	88.0
No	12	12.0
Usage of PPEs		
Yes	81	81.0
No	19	19.0
Frequency of PPE usage	(n=81)	
Always	12	14.9
Sometimes	21	25.9
Rarely	18	22.2
When available	9	11.1
Only when a supervisor is present	21	25.9

The pulmonary function test of study participants is represented in Table 4. The mean ± SD FEV1 of paint factory was 2.49±0.69 (L/sec) compared to 3.72±0.71 (L/sec) among non-PFWs. This difference was statistically significant (p < 0.0001).

**Table 4:** Pulmonary function test of study participants PFW - paint factory workers, NPFW - non-paint factory workers, FEV1 - Forced expiratory volume, FVC - Forced vital capacity, t - Student t-test, P-values <0.05 are significant (*)

Spirometry parameter	PFW, Mean ± SD	NPFW, Mean ± SD	Test statistics	P-value
FEV_1_ (L/sec)	2.49±0.69	3.72±0.71	t= -14.285	*<0.0001
FVC (L/sec)	3.63±0.84	4.27±0.95	t= -6.949	*<0.0001
FEV_1_/FVC %	68.20±7.89	88.35±9.60	t=-18.385	*<0.0001
Restrictive (n = 43)	(n = 34)	(n = 9)		
FEV_1_ (L/sec)	2.70±0.62	3.62±0.59	t = -4.037	*<0.0001
FVC (L/sec)	3.60±0.84	5.14±0.83	t = -4.910	*<0.0001
FEV_1_/FVC %	75.01±2.55	77.17±2.46	t = -5.364	*<0.0001
Obstructive (n = 72)	(n = 59)	(n = 13)		
FEV_1_ (L/sec)	2.24±0.59	3.65±0.52	t = -7.965	*<0.0001
FVC (L/sec)	3.56±0.85	5.37±0.79	t = -7.064	*<0.0001
FEV_1_/FVC %	62.65±4.56	68.06±1.81	t = -4.188	*<0.0001

The mean ± SD FVC of paint factory was 3.63±0.84 (L/sec) compared to 4.27±0.95 (L/sec) among non-PFWs. This difference was statistically significant (p < 0.0001). The mean ± SD FEV1/FVC of PFWs was 68.20±7.89 compared to 88.35±9.60. This difference was statistically significant (p < 0.0001).

**Figure 1:**
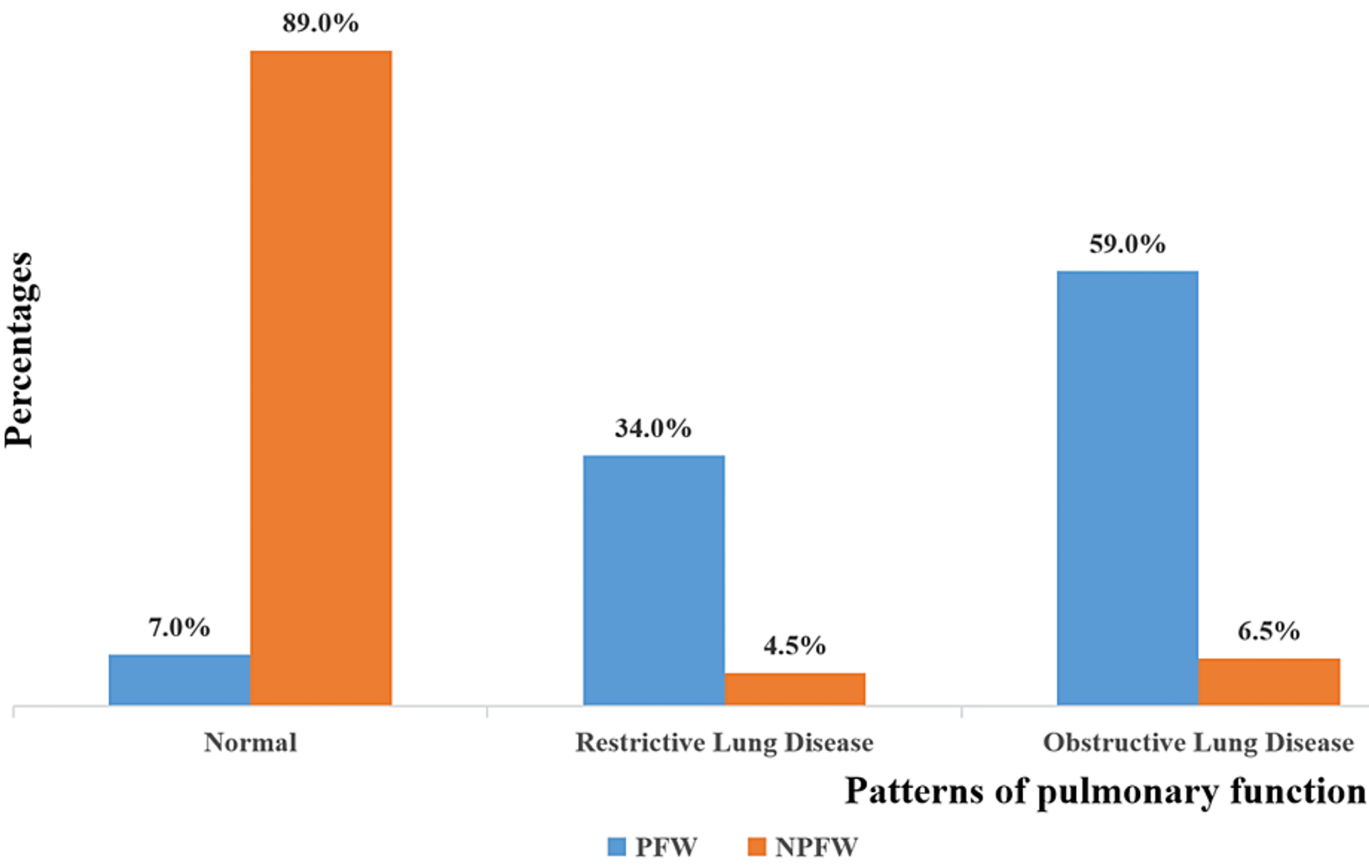
Patterns of pulmonary function test among study participants PFW- paint factory workers, NPFW- Non-paint factory workers, Chi-square test (ꭓ2) = 189.731, P-values is <0.0001 which is significant

The patterns of pulmonary function tests among study participants are shown in Figure 1. Most NPFWs 178 (89.0%) had normal pulmonary function tests. Regarding the spirometric abnormality in PFWs, most of them (59.9%) had obstructive lung diseases while others had restrictive (34.0%) and normal findings (7.0%).

The socio-demographic characteristics and spirometric findings among paint factory study participants is shown in Table 5. Thirteen (86.7%) paint factory study participants aged ≥50 years had restrictive lung disease compared to two (13.3%) in a similar age group. The association between age group and type of lung abnormality was not statistically significant (p=0.044).

**Table 5:** Relationship between sociodemographic characteristics and spirometric findings among paint factory study participants PPE - Personal protective equipment, F - Fishers Exact Test, ꭓ2 - Chi-square test, P-values <0.05 are significant

Variable	Restrictive lung abnormality group	Obstructive lung abnormality group	Test statistics	P-value
	Freq. (%) (n=74)	Freq. (%) (n=26)		
Age group (Years)				
< 20	1 (100.0)	0 (0.0)	F= 5.437	0.044
20-29	20 (66.7)	10 (33.3)		
30-39	16 (69.6)	7 (30.4)		
40-49	24 (77.4)	7 (22.6)		
>50	13 (86.7)	2 (13.3)		
Sex				
Male	43 (75.4)	14 (24.6)	ꭓ^2^= 0.143	0.819
Female	31 (72.1)	12 (27.9)		
Educational level				
No formal	0 (0.0)	1 (100.0)	F= 3.837	0.257
Primary	15 (83.3)	3 (16.7)		
Secondary	35 (72.9)	13 (27.1)		
Tertiary	24 (72.7)	9 (27.3)		
Duration of employment				
≤5 years	32 (71.1)	13 (28.9)	ꭓ^2^= 5.824	0.047
6-10 years	30 (73.2)	11 (26.8)		
>10 years	12 (85.7)	2 (14.3)		
PPE usage				
Yes	60 (74.1)	21 (25.9)	F= 0.001	1.000
No	14 (73.7)	5 (26.3)		
Frequency of PPE usage (n=81)				
Always	8 (66.7)	4 (33.3)	F= 1.776	0.787
Sometimes	16 (76.2)	5 (23.8)		
Rarely	15 (83.3)	3 (16.7)		
Only when available	6 (66.7)	3 (33.3)		
Only when the supervisor present	15 (71.4)	6 (28.6)		

Forty-three (75.4%) male paint worker study participants had restrictive lung disease compared to 14 (24.6%) with obstructive disease, while 31 (72.1%) female paint worker study participants had restrictive lung disease compared to 12 (27.9%) with obstructive disease. The association between gender and lung abnormality was not statistically significant (p=0.819).

Of more paint workers with a restrictive disease, 15 (83.3%) had a primary level of education while the lowest proportion, 24 (72.7%) of paint workers with restrictive disease was seen among those who had a secondary level of education. The association between level of education and lung abnormality was not statistically significant (p=0.257).

Thirty-two (71.1%) paint workers who had spent less than five years working had restrictive lung disease compared with 12 (85.7%) of the paint workers who had been working for 10 years or more. The association between duration of work and lung abnormality was statistically significant (p=0.047).

Sixty (74.1%) paint workers with restrictive disease had used PPEs while 21 (25.9%) with obstructive disease had also used PPEs. The association between PPE usage and lung abnormality was not statistically significant (p=1.000).

Fifteen (83.3%) paint workers who had restrictive lung disease used PPEs rarely while eight (66.7%) paint workers with obstructive always used PPEs work. The association between frequency of PPE use and lung abnormality was not statistically significant (p=0.787).

More paint workers with a restrictive disease, 26 (81.2%) worked in the manufacturing section compared with those nine (64.3%) who worked in the quality assurance unit. The association between factory section and lung abnormality was statistically significant (p=0.021).

## Discussion

A total of 300 participants were recruited for this study. The number of PFW was 100 while that of NPFW was 200. This study assessed lung impairment from spirometric findings among PFWs in Delta State, Nigeria. Results from the study aptly showed that PFWs who are chronically exposed to paint had more abnormalities with obstructive patterns more predominant than restrictive spirometric findings. In the index study, PFW and NPFW showed no significant difference in terms of sociodemographic characteristics. In a similar study done in Ile-Ife Nigeria, where 120 paint workers were recruited into the study, it showed that there was no significant difference in the sex, age, height, and weight of the study population [11]. In this study, the sex distribution had more males than females with a male-to-female ratio of 1.3:1.This was probably because working in paint factories involved more manual labor - a job description that was more suited for males than females. Similarly, in a study conducted by Awodele et al. [12] in Lagos Nigeria, a total of 400 randomly selected PFWs with sex distribution showed a predominantly male setting than a female setting thereby having an approximate male/female ratio of 2:1.24.

The mean spirometric values in this study showed that there was a significant reduction in FEV1, FVC, and FEV1/FVC of the PFW when compared to the NPFW (p < 0.05). The pattern of spirometric findings observed showed that only 7% of those in the PFW group had normal spirometric findings while as high as 59.0% had obstructive findings and 34.0% had restrictive patterns. On the other hand, most of the participants in the NPFW group had normal spirometric findings (89.0%) with restrictive spirometric findings being seen in the least proportion of participants in the group 4.5% and obstructive pattern observed in 6.5%. The rate of abnormalities was similar and can be comparable to findings conducted by Rahhal et al. [13] in Palestine which showed both (68% restrictive and 14% obstructive) [13]. Jabbar et al. [2] in Basrah City, South of Iraq, also showed obstructive (34.5%) and restrictive (24.1%) spirometric findings. Another study conducted in India by Chattopadhyay showed that 25.83% of the workers had obstructive while 21.19% of PFWs had restrictive spirometric findings [14]. Similar findings to the index study were observed by Onesmo et al. where 28% were found to have normal spirometry, about 28% were also found with only airway obstruction, 12% were found with only lung restriction whereas 32% were found with both airway obstruction and lung restriction problems [15]. In addition, paint industry workers of West Bengal also had normal spirometry in 34.61%, a restrictive spirometric finding in 23.07% while 30.76% had obstructive airways and 11.53% had combined [16]. Studies from other occupations such as petrol pump workers that are exposed to VOCs like toluene, benzene, and xylene also have restrictive spirometric findings where Ramadhany et al. reported abnormalities in 42.3% of subjects [17]. The inhaled VOCs get deposited in the lungs, causing an inflammatory reaction leading to lung fibrosis, faulty oxygen diffusion, and abnormal lung functions (restrictive) [11,12,18,19]. The changes in the elasticity and viscosity of the mucous affect its clearance and result in a luminal mucous plug, which is responsible for obstruction to the airflow leading to a decrease in FVC, FEV1, and FEV1/FVC ratio [20].

Studies have shown that long-term exposure to paint fumes is associated with a higher prevalence and risk of a significant decline in spirometric findings [2]. This decline showed a more significant difference among the paint workers who were exposed to paints for more than 10 years, thereby indicating the significant role of duration of exposure in this study. This is in keeping with Saraei et al. [18]. where exposures of greater than 10 years indicate a significant role of duration of exposure in causing a decline in spirometric findings [18]. The restrictive and obstructive spirometry findings identified in this study were associated with years of exposure to paint but not with the fraction of time spent while producing the paint. These can also be attributed to other factors that could work together besides the toxic effect of the VOC of the paint to exacerbate the impairment in the pulmonary function tests, lack of awareness about the effect, and non-usage of suitable PPE [2,6]. Workers at the paint factory were aware of the use of personal protective equipment (88%) but their usage rate was very low as only 12% used their PPE always. The same results were found by Onesmo et al. [15] which showed that about 75% of workers had some kind of personal protection provided but low usage [15]. In other factory sites, the protective equipment was available but was not being used because they reduced their efficiency as the PPE did not allow the workers to breathe smoothly while some would use the PPE only when their supervisors were around. These showed that they had low knowledge concerning the toxic effects of paints [11,15,21]. The lack of enforcement of these facilities at the working sites is another issue of consideration that needs attention [21].

The difference in the percentage of usage of PPE could be attributed to the fact that despite providing PPE by the provider, some were not using theirs because they felt they were now “experts” in their work and so they did not need them. These concluded that they had low knowledge concerning the effects of painting [21]. Another interesting fact is that even though PFW knows very well about the hazards associated with the inhalation of painting materials, they felt that just drinking fresh milk before and after work was the solution to protect them from respiratory problems. The same results were obtained by Adei et al. [21], where the workers believe the use of soda water or milk as a body detoxifying agent after considerable exposure [21]. This may be because of their low level of knowledge and as they do not receive any training concerning the importance of using PPE.

Recommendations

As already outlined, safety measures should be employed to reduce the health hazards for workers, this is done by ensuring they wear appropriate respiratory and protective equipment, prevent spillages, and ensure airborne concentrations of vapor do not exceed the Permissible Workplace Exposure Standard. We also recommend training and education of workers on the effects of substances they are being exposed to and not just with vague awareness with so many misconceptions.

Limitations

This study is limited firstly, following the over-pollution of Delta State as a result of gas flaring and other forms of air pollutants, some participants may already have some forms of respiratory impairment. Secondly, being a cross-sectional study, this study may be susceptible to survivor bias as there is a possibility that individuals with symptoms were more willing to participate than those without symptoms, therefore, the subject selection was not truly random.

## Conclusions

The results from this study have shown that people exposed to VOCs, as emitted from paint fumes, have spirometric readings that are suggestive more of obstructive than restrictive patterns. The lack of use of PPE and a longer duration of exposure was associated with a higher prevalence of abnormal spirometric findings observed among the study group. The non-use of protective equipment was not a result of the decreased awareness; however, it was due to poor knowledge of the effect paint exposure might have on the respiratory system as such, there were no effective measures to ensure their use.

## Figures and Tables

**Figure 2:**
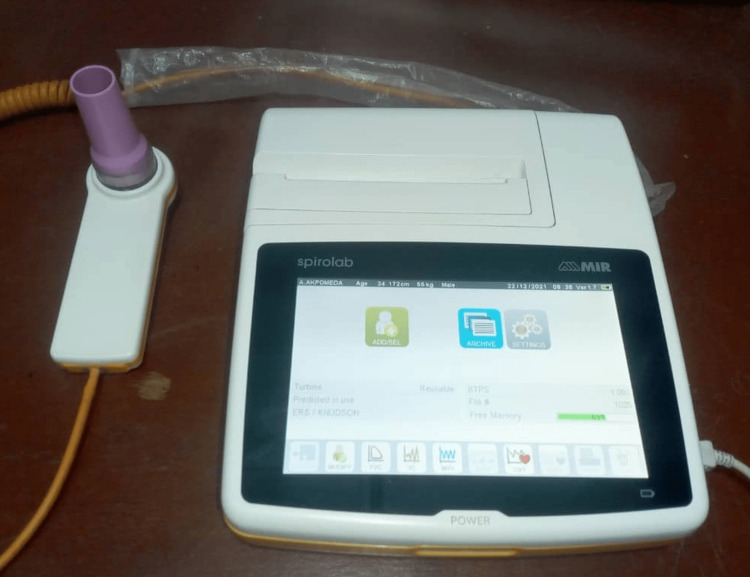
MIR Spirolab Spirometer

## Appendices

**Table 6:** Questionnaire: The pattern of respiratory symptoms and spirometric findings amongst paint factory workers in Delta State

Non-factory worker ( )	Factory worker ( )		Factory site:	
1. Age in years:		2. Sex: Male ( ) Female ( )		
3. Tribe: ……….	4. Occupation……………..			5. Duration of employment…..……
6. Description of work				
7. Marital status: single ( ) married ( ) separated ( ) widowed ( ) Divorced ( )				
8. Religion: none ( ) Christianity ( ) Islam ( ) Traditional religion ( ) Other ……				
9. Highest level of education: None ( ) Primary ( ) Secondary ( ) Tertiary ( )				
10. Are you aware of any personal protective equipment/devices used in relation to your work? Yes ( ) No ( ) If yes, please specify them_____				
11. Do you use any of these PPE provided while at work? Yes ( ) No ( )				
12. If yes, how often? Always when I am working ( ) Sometimes when I feel like ( ) Rarely ( ) Only when it is available ( ) Only when my supervisor is present ( ) Never even if it is available ( )				
13. Spirometry Test				
	Actual		Predicted	%predicted
FEV_1_, (L)				
FVC (L)				
FEV_1_/FVC %				
PEFR (L/min)				
FEF25–75%				
